# Alzheimer's Disease Biomarkers, Cognitive Reserve, and Cognition in MCI and Cognitively Unimpaired Older Adults: A structural equation model

**DOI:** 10.1002/alz70856_104874

**Published:** 2026-01-07

**Authors:** Jordan P. Sergio, Louisa I. Thompson, Ashley N. Price, Jennifer Strenger, Megan Stradtman, Sarah Benjamin, Stuart Sinoff, Peter J. Snyder, Jessica Alber

**Affiliations:** ^1^ Butler Hospital Memory and Aging Program, Providence, RI, USA; ^2^ University of Rhode Island, Kingston, RI, USA; ^3^ Alpert Medical School of Brown University, Providence, RI, USA; ^4^ Bryn Mawr College, Bryn Mawr, PA, USA; ^5^ Butler Hospital, Providence, RI, USA; ^6^ BayCare Health, Clearwater, FL, USA; ^7^ George & Anne Ryan Institute for Neuroscience, Kingston, RI, USA

## Abstract

**Background:**

While brain imaging techniques such as amyloid PET scans are sensitive and specific biomarkers of Alzheimer's disease (AD) neuropathology, they are not scalable from a public health perspective. Other biomarkers, including blood and retinal biomarkers, are proposed as suitable alternatives which correlate with amyloid PET burden. Additionally, individual differences in resiliency to AD neuropathology may be explained by the cognitive reserve theory. Higher cognitive reserve, measured by proxies including education and occupational attainment, allows individuals to maintain optimal cognitive functioning for longer despite the presence of AD neuropathology. Indeed, education and occupational complexity reduce the risk of developing AD. Therefore, it is imperative to better understand the relationship between biomarkers of AD and brain resiliency. The current study examines associations between biomarkers of AD (blood and retinal layer thickness), cognition, and proxies of cognitive reserve.

**Method:**

Participants (*n* = 115) were older adults aged 55‐80, including 15 MCI patients (MoCA>19, <24, CDR=.5) and 100 cognitively unimpaired (CU) (MoCA>=26, CDR=0) recruited as part of the Atlas of Retinal Imaging in Alzheimer's Study. A structural equation model was run to elucidate relationships between several constructs including latent variables of cognition, which included measures of attention, processing speed, visuospatial construction, and executive function, AD pathology burden, as indicated by plasma biomarkers, and cognitive reserve (indicators including years of education and occupational complexity). Retinal layer thickness of the ganglion cell layer complex (GCC) was also measured. Regressions, accounting for age, included AD pathology burden and GCC thickness predicting cognition, cognitive reserve predicting GCC thickness, and cognitive reserve predicting AD pathology burden.

**Result:**

Age was a significant predictor of AD pathology burden and average GCC thickness. AD pathology burden was a significant predictor of cognition while accounting for age. No other predictors in the SEM were significant.

**Conclusion:**

A latent variable including blood biomarkers ptau217, ptau181, and neurofilament light chain to represent AD pathology burden predicted cognition. This adds to mounting evidence that blood biomarkers accurately predict cognitive changes in CU and MCI older adults. Delineating whether these effects were driven by MCI or high‐risk CU may be a future direction with larger sample sizes.

